# Editorial: 2022 in review: food allergy

**DOI:** 10.3389/falgy.2024.1429903

**Published:** 2024-05-20

**Authors:** Angel Mazon, Antonella Muraro, Alexandra F. Santos, Ronald van Ree

**Affiliations:** ^1^ Unit of Pediatric Allergy and Pneumology, Research Institute La Fe, University and Polytechnic Hospital La Fe, Valencia, Spain; ^2^ Food Allergy Referral Centre Padua, University Hospital, Padua, Italy; ^3^ Department of Women and Children’s Health (Paediatric Allergy), Faculty of Life Sciences and Medicine, School of Life Course Sciences, King’s College London, London, United Kingdom; ^4^ Children’s Allergy Service, Evelina London Children’s Hospital, Guy’s and St Thomas’ Hospital, London, United Kingdom; ^5^ Peter Gorer Department of Immunobiology, School of Immunology and Microbial Sciences, King’s College London, London, United Kingdom; ^6^ Department of Experimental Immunology, Amsterdam University Medical Centers, Amsterdam, Netherlands

**Keywords:** milk allergy, hydrolyzed formula, inorganic nanoparticle, perinatal exposure, food allergy risk, dining industry, DNA vaccine, Gun-delivered vaccination

**Editorial on the Research Topic** 2022 in review: food allergy

The increasing prevalence of food allergies constitutes a public health problem (1, 2). Patients with severe food allergy are at risk of severe reactions and even death. Those with milder allergies, although they do not run the same risk, have impaired quality of life (3), due to limitations in daily life, and incur in higher costs (4), both in terms of personal expenses and health care, for diagnosis, monitoring and treatment. This Research Topic of Frontiers in Allergy offers insights into factors influencing the onset of food allergy, current therapies for cow's milk allergy in infants, novel modes for administration of vaccines for established food allergy, and impact on social life of patients with food allergies.

A classic statement is that food allergy depends on the interaction between genetic and environmental factors. There is general agreement that genetic changes take a long time and do not explain the food allergy epidemics of the last 40–50 years. Therefore, the focus of research has been on the changes of modern life that occurred in parallel with the rise in food allergy. These changes are probably multifactorial. Issa et al. review the role of inorganic nanoparticles during perinatal life. These agents are very ubiquitous and can be found in many foods, where they are added directly or indirectly from contact materials during storage, manufacturing and packaging. These nanoparticles have beneficial properties for food safety but, if ingested by the mother, they can reach the fetus or newborn through the placenta or breastfeeding. They can have effects on the bacterial microbiome, on intestinal permeability and, in addition, they are absorbed and interact with the immune cells of the GALT, with deleterious effects on Treg cells and B cells. All of these potential mechanisms of nanoparticles can favor the development of allergy and deserve to be taken into account.

Cow's milk allergy in infants is treated by avoiding intact milk proteins and administering alternative formulas. Most used are the extensively hydrolyzed formulas, based on casein and/or whey. There are several brands, with different peptide size distribution profiles, and some have other added functional ingredients. The review by Goh et al. compares several formulas and highlights the main effects of their components. Several mechanisms are described, the interaction with T and B cells, the induction of tolerogenic cytokines and the restoration of the intestinal barrier. Likewise, the effect of prebiotics, probiotics and their different combinations with different types of formula is also evaluated. Thus, the different results found lead the authors, although acknowledging that this is not a systematic review, to conclude that not all of these formulas have equivalent effectiveness.

The treatment for food allergy used to consist solely of avoiding the offending food and reassessing over time for spontaneous tolerance, which is common in young children, but not in older children or adults. In recent years, “active” treatments have been used, such as oral tolerance induction or the use of vaccines. In the original study by Smeekens et al., the effect in mice of Gene Gun-delivered DNA vaccines targeting crustacean or walnut/pecan allergens was evaluated. In this study, there are several novel aspects to highlight. First, the use of DNA plasmids instead of molecular allergens. Second, the effective mode of delivery, the novel Gene Gun-delivered administration. Finally, the addition of interleukin IL-12 had significant additional effects only in the CC027/GeniUnc strain of mice, prone to severe allergic reactions similar to those in humans. These vaccines were able to induce IgG antibodies, with better results for crustacean allergens, expressed more intensely than some of the important storage proteins of walnuts and pecans.

One of the factors with negative impact on quality of life of food allergic patients is the practical challenges of having meals outside of the home, especially when eating non-prepackaged food. In the review by Stankovich et al., risk assessment and possible solutions thereof are discussed. Allergen quantification methods and legal regulations for prepackaged foods, which constitute the safest part for food allergic patients, are described for several countries. Less safe environment for food allergic patients are restaurants and other retail food establishments. In this context, there is a lack of homogeneous national regulations in the USA or across European countries, and different initiatives by state or local authorities are reviewed. Finally, new labeling tools are described, including allergen quantification and reaction risk assessment, which could help improve the safety of allergic patients.
